# Graves' Disease as a Manifestation of Immune Reconstitution in HIV-Infected Individuals after Initiation of Highly Active Antiretroviral Therapy

**DOI:** 10.1155/2011/743597

**Published:** 2011-07-25

**Authors:** Samad Rasul, Robert Delapenha, Faria Farhat, Jhansi Gajjala, Syeda Mehreen Zahra

**Affiliations:** Division of Infectious Diseases, Department of Internal Medicine, Howard University Hospital, Washington DC, USA

## Abstract

Graves' disease after the initiation of highly active antiretroviral therapy (HAART) in certain HIV-1-infected individuals has been described as an immune reconstitution inflammatory syndrome (IRIS). This phenomenon should be suspected in individuals who present with clinical deterioration and a presentation suggestive of hyperthyroidism despite good virological and immunological response to HAART. Signs and symptoms of hyperthyroidism may be discrete or overt and typically develop 8–33 months after initiating therapy. One to two percent of HIV-infected patients can present with overt thyroid disease. Relatively few cases of Graves' IRIS have been reported in the literature to date. We describe four cases of Graves' IRIS in HIV-infected patients who were started on HAART therapy.

## 1. Introduction

The beginning of the highly active antiretroviral therapy (HAART) era around 1995 signaled a paradigm shift in the clinical outcome of patients infected with HIV as reflected by a significant improvement in overall survival [1]. Patients with HIV infection, who are on HAART, achieve restoration of previously compromised immune function, resulting in decreased mortality and morbidity from opportunistic infections [2]. However, a minority of patients might experience a paradoxical clinical decline as a result of immune restitution [2]. This phenomenon occurs by virtue of restoration of the capacity to mount an inflammatory response against both infectious and noninfectious antigens [3], hence the term immune reconstitution inflammatory syndrome (IRIS). IRIS occurs most commonly as a result of reactivation of infections such as *Mycobacterium avium* complex, *Mycobacterium tuberculosis*, *Cryptococcus neoformans,* or cytomegalovirus and tends to occur between a few weeks and several months after initiating HAART, corresponding with CD4 positive memory cell repopulation [4].

The development of a variety of autoimmune diseases has been reported in patients infected with HIV. Autoimmunity may occur due to the loss of immune competence but may also appear after commencement of HAART [5]. Indeed, autoimmunity as a consequence of immune reconstitution has been recognized as an unfavorable event in HIV-1-positive individuals. Graves' disease resulting from immune restoration has had relatively recent recognition and might be viewed as a consequence of organ-specific autoimmunity during the late period of T-cell repopulation, specifically of CD4 positive naïve cells [6]. We report four cases of Grave's IRIS in our practice. The detailed demographics and HIV-related history is summarized in Table 1 and thyroid-related workup and management in Table 2.

**Case 1**

A 34-year-old African American male with a CD4 T-cell count of 59 cells/*μ*L and HIV RNA level >750,000 copies/mL was started on stavudine, abacavir, lamivudine and efavirenz. Forty-four months later, he presented with weight loss, alopecia, diarrhea, palpitations, lid lag, and thyromegaly. Thyroid function tests revealed elevated free T4 and thyroid peroxidase antibody (TPO-Ab) levels, with undetectable thyroid stimulating hormone (TSH) levels. A thyroid ultrasound revealed multinodular changes with diffuse enlargement of the gland. He was treated with methimazole and ultimately I-131 radioablation with complete resolution of symptoms.

**Case 2**

A 42-year-old African American male with a nadir CD4 T-cell count of 89 cells/*μ*L and HIV RNA level of 205 copies/mL initiated a regimen of lamivudine, zidovudine, and efavirenz with good response. Fifty-three months later, he presented with weight loss, tremor, exophthalmos, and an enlarged thyroid gland. Thyroid function tests were consistent with hyperthyroidism with an enlarged thyroid gland on ultrasound. Thyroid scintigraphy demonstrated the elevated uptake of I-123. He was managed successfully with methimazole and atenolol.

**Case 3**

A 39-year-old African male presented with a CD4 T-cell count of 59 cells/*μ*L and an undetectable HIV RNA level. He was treated with tenofovir/emtricitabine/efavirenz. Within thirty-one months, he presented with palpitations, tremors, fatigue, and weight loss. The thyroid gland was enlarged. Thyroid function tests revealed an undetectable TSH with high free T4 and antithyroglobulin antibodies. Graves' disease was managed with metoprolol and propylthiouracil with complete clinical response.

**Case 4**

A 43-year-old African American female had an initial CD4 T-cell count of 29 cells/*μ*L and HIV RNA level of 252, 984 copies/mL. She began tenofovir/emtricitabine/efavirenz and tolerated HAART well. Nineteen months later, she experienced tremor, weight loss, and diarrhea with reduced appetite. Her thyroid gland was enlarged and pulsatile, and she had exophthalmos with a lid lag. Her condition was managed with methimazole and metoprolol.

## 2. Discussion

Autoimmunity has been described in untreated HIV infection; however, apart from immune thrombocytopenia, reports of organ-specific autoimmunity are few [2]. Rheumatoid factor, ANA, and antiphospholipid antibodies may be found in HIV-infected individuals without clinical correlation [7]. Graves' disease has in recent times been described in the literature as a documented IRIS among HIV-infected patients. Graves's disease usually occurs after a rise in the CD4 T-cell count; however, its unique nature is typified by its late presentation, usually 8–33 months after starting HAART [4, 8, 9]. 

Graves' IRIS was initially described by Gilquin et al. in 1998 when they described 3 cases [10]. Briefly following this, Jubault et al. studied these cases in detail in conjunction with 2 other cases [7]. Since the initial description, the number of cases reported has been low. Crum et al. provided a comprehensive review of 28 cases known at the time of description [11]. In our patients, the median duration of diagnosis of Graves' IRIS after the initiation of antiretroviral therapy was 37.5 months (range 19–53 months) compared to a range of 14–22 months described in initial reports [2, 7]. Our patients were predominantly black males (75%) in comparison with previously available data where 19/28 (67.8%) were predominantly black females [8, 10].

The median CD4 T-cell nadir in our patients was 59 cells/*μ*L (range 29–89 cells/*μ*L), and the median rise in CD4 T-cell count until the time of developing symptoms was 357.5 cells/*μ*L (range 191–448 cells/*μ*L). All patients had drastically reduced or undetectable viral loads at the time of diagnosis of Graves' IRIS. Like all other cases described in the literature, none of our cases had a pre-existing thyroid condition. In addition, there was no documented history of other autoimmune diseases. None of our patients was coinfected with hepatitis C or ever treated with interferon. We, therefore, inferred that the clinical picture of our patients was consistent with Graves' IRIS. 

Reconstitution Graves' disease has been described to occur in 3 clinically distinct settings [8]. Bone marrow transplantation from a donor who has Graves' disease may cause this disease to surface in the recipient. Alemtuzumab treatment for multiple sclerosis may also generate the development of Graves' disease in up to a third of patients. Finally, reconstitution Graves' disease may follow HAART in HIV-1 infected individuals. Chaotic immunoregulation due to graft-versus-host disease might play a pathogenic role in bone marrow transplant recipients, whereas naïve CD4 T-cell expansion has been hypothesized as the major causative mechanism in the Alemtuzumab and HAART-induced reconstitution Graves' disease [8]. 

In the first 2-3 months following HAART, HIV replication is suppressed, and the release of memory T cells from inflamed lymphoid tissues leads to a rapid increase in the previously low CD4 T-cell numbers [4, 8]. After this period, naive CD4 T-cell numbers slowly improve as a result of repopulation and new thymic production. Graves' disease may also be associated with Interferon Alpha (IFN*α*) used to treat hepatitis C and Interleukin 2 (IL-2) therapy in the setting of HIV [3, 9]. Earlier studies found that TPO antibodies and TSH receptor antibodies appeared after CD4 T cells had dramatically increased on HAART, whereas they were repeatedly absent prior to HAART [7]. The occurrence of Graves' disease in some reports was closely associated with the rise of TSH receptor antibodies [6, 7].

Disorders of thyroid function are comparatively common in the HIV population. Among individuals infected with HIV, 1%-2% might experience clinically apparent thyroid disease, and 35% may have subtle defects in thyroid function test findings [12]. In addition to Graves' disease, numerous other thyroid disorders have been noted in HIV including overt hypothyroidism, subclinical hypothyroidism, sick euthyroid syndrome, and thyroiditis caused by opportunistic infections or infiltrative processes [8, 12]. It is largely unknown why only certain individuals develop Graves' disease as part of an immune reconstitution process although various polymorphisms in different cytokine and MHC genes have been identified or hypothesized in different studies [6–9].

Whereas the pathogenic role of specific HAART agents has not yet been validated, there has been speculation about this possibility over the years. Most studies, however, argue that Graves' IRIS is more of a consequence of immune reconstitution rather than a matter of how such reconstitution is achieved. Recently, a large single center cohort study described thyroid disorders in HIV patients over a course of 11 years [13]. In this cohort involving 2437 individuals, the clinical prevalence of hyperthyroidism was 1.01%, and that of hypothyroidism was 1.2%. Hypothyroidism was most commonly associated with a protease inhibitor-based regimen and hyperthyroidism with non-nucleoside reverse transcriptase inhibitors, particularly efavirenz [13]. All the four patients we have described were also on a regimen containing efavirenz. Although this might reflect the frequent use of efavirenz in initial HAART regimen, a direct role of this agent in the pathogenesis of autoimmune thyroid disease in HIV-infected individuals can be speculated. 

## 3. Conclusion

Graves' IRIS should be suspected if an HIV-infected individual presents with weight loss or other signs of hyperthyroidism despite adequate and sustained immunological and virological control. In HIV patients, measurement of TSH level is appropriate for those with signs or symptoms suggestive of thyroid dysfunction. There may be a need to evaluate the role of specific antiretroviral agents in the pathogenesis of Graves' IRIS. Longitudinal studies are required to further elucidate the effect of HAART in general on thyroid antibodies and to possibly delineate clinical risk factors or predictors for developing Graves' IRIS.

## Figures and Tables

**Table 1:** Demographic, immunological and virological data for the cases described.

No.	Year	Age	Sex	Race	HIV diagnosis	HAART start	Baseline CD4 count (cells/*μ*L)	CD4 count at symptoms (cells/*μ*L)	HIV RNA change (copies/mL)	HAART regimen
(1)	2003	34	M	African American	March 1993	June 1999	59	341	>750,000 to 775	Stavudine
										Abacavir
										Lamivudine
										Efavirenz

(2)	2007	42	M	African American	December 2002	December 2002	89	537	205 to <50	Lamivudine
										Zidovudine
										Efavirenz

(3)	2007	39	M	African	April 2005	April 2005	59	250	<50 to <50	Tenofovir
										Emtricitabine
										Efavirenz
										Tenofovir

(4)	2008	43	F	African American	March 2006	October 2006	29	462	252,984 to <50	Emtricitabine
										Efavirenz

**Table 2:** Thyroid workup and management (FT4: free T4, TSH: thyroid stimulating hormone, TPO-Ab: thyroid peroxidase antibody, TBG-Ab: thyroglobulin antibody, PTU: propylthiouracil).

No.	Thyroid Work Up	Time till symptoms	Symptoms	Previous thyroid condition	HCV coinfection	Treatment	History of autoimmune disease
(1)	FT4: 7.43 ng/dL TSH < 0.01 MIU/mL TPO-Ab: 46.7 IU/mL TBG-Ab < 1 IU/mL	44 months	Thyromegaly, lid lag, weight loss, alopecia, diarrhea, and tachycardia	No	No	Methimazole I-131 radioablation	No

(2)	TSH < 0.01 MIU/mL FT4: 3.3 ng/dL Diffuse uptake on thyroid scan	53 months	Tremor, weight loss, exophthalmos, and thyromegaly	No	No	Methimazole atenolol	No

(3)	TSH < 0.004 MIU/mL T4: 12.2 ng/dL TBG-Ab: 40 IU/mL	31 months	Weight loss, tremor, exophthalmos, lid lag, and fatigue	No	No	PTU metoprolol	No

(4)	Not available	19 months	Tremors, weight loss, loss of appetite, thyromegaly, and exophthalmos	No	No	methimazole metoprolol	No
